# Conditional reciprocal stressor–strain effects in university students: a cross-lagged panel study in Germany

**DOI:** 10.1038/s41598-024-57486-0

**Published:** 2024-03-23

**Authors:** Jennifer L. Reichel, Lina M. Mülder, Pavel Dietz, Sebastian Heller, Antonia M. Werner, Markus Schäfer, Lisa Schwab, Stephan Letzel, Thomas Rigotti

**Affiliations:** 1 Institute of Occupational, Social and Environmental Medicine, University Medical Center of the University of Mainz, Mainz, Germany; 2 Department of Work, Organizational, and Business Psychology, Institute for Psychology, Johannes Gutenberg University, Mainz, Germany; 3 Department of Psychosomatic Medicine and Psychotherapy, University Medical Center of the University Mainz, Mainz, Germany; 4 Department of Communication, Johannes Gutenberg University, Mainz, Germany; 5 Department of Sport Science, Johannes Gutenberg University, Mainz, Germany; 6 Leibniz Institute for Resilience Research, Mainz, Germany; 7 Department of Culture, Media and Psychology, Macromedia University of Applied Sciences, Frankfurt, Germany

**Keywords:** Stressor effect, Strain effect, University student, Emotional exhaustion, Depression, Well-being, Psychology, Psychiatric disorders

## Abstract

University students worldwide are facing increasing mental health challenges. Traditional stress models, like the Job/Study Demand-Resources Model, link stressors directly to strain. Yet, recent studies suggest the influence of strain on stressors may be even stronger. Our research explored these reciprocal dynamics among university students, considering social support and mindfulness as potential moderators. We conducted a two-wave panel study with 264 university students. We ran separate cross-lagged panel structural equation analyses for three key health outcomes—emotional exhaustion, depression, and well-being—each paired with perceived study stressors, specifically workload and work complexity. Findings revealed significant stressor and strain effects, with social support notably moderating the impact of emotional exhaustion on workload. These insights challenge traditional stress theories and underscore the importance of mental health support and effective stress management strategies for students, emphasizing the need for proactive mental health initiatives in academic environments.

## Introduction

Many university students around the world struggle with mental health issues. During the COVID-19 pandemic, burnout, depression and loneliness have even increased among students^1^. Several authors have reported the alarming impact of the pandemic on students’ mental health^2^. According to a recent meta-analysis, the pooled prevalence of depressive symptoms among students in 2020 was 34%^3^. Research prior to the pandemic reported lower prevalence rates, such as 30% in a systematic review by Ibrahim et al.^4^. Also in comparison to prevalence rates in the general population (28.8% during the COVID-19 pandemic, 25.3% before the pandemic^5^), students can be considered a highly vulnerable group. The demanding academic environment is a significant contributor to this heightened risk, as it exacerbates the prevalence of mental disorders among students^6,7^. This situation underscores the critical need for effective health promotion strategies in university settings, as outlined in the Okanagan Charter^8^. A key focus of these strategies should be the identification and mitigation of stressors that adversely affect student mental health.

To build a deeper evidence base on the causes and consequences of mental health problems, Robins et al.^9^ emphasized that studies on student mental health need to be informed by appropriate theories, such as the Job Demand-Resources (JD-R)^10^. The JD-R model as well as its adaption to the student context (Study-Demands-Resources Model^6^) assumes that demands are resource-consuming, require effort to cope with, and are thus positively associated with psychological strain and adverse health outcomes. Recent meta-analytic evidence has shed new light on this causal relationship, by showing that the causal effects of strain on stressors are even stronger than those of stressors on strain^11^.

The current study aims to examine reciprocal stressor-strain effects in a student sample, which has rarely been pursued in previous research. In addition, we add social support and mindfulness as potential moderators, which have been studied primarily for stressor, but not strain effects (even in the work context). We chose social support and mindfulness as moderators to represent both an external element (social support) and an internal one (mindfulness), aiming to examine how these factors might ease the reciprocal impact between stressors and strains. This dual focus offers insights into both individual-focused and structural prevention strategies, enhancing our understanding of stress management. Hobfoll^12^ argued that social support broadens an individual's resource pool and strengthens existing ones, thus fostering a cycle of resource gain. Social networks provide crucial information and guidance, helping students tackle academic challenges and make informed decisions, thereby lessening stress related to uncertainty and problem-solving.

Mindfulness, understood as a state of nonjudgmental awareness of present experiences^13,14^, is increasingly recognized for its relevance in academic contexts^15^. This is especially pertinent for university students, offering insights into enhancing their mental well-being and academic performance. We posit that mindfulness is a vital moderator for university students regarding how stressors affect their health and well-being and vice versa. Emotional strain often leads to counterproductive rumination, but mindfulness helps redirect energy from these patterns to creative and resourceful activities^16,17^. Thus, more mindful students are likely to use their negative emotions in ways that encourage resource acquisition. As our research explores the two-way relationship between perceived study demands and health outcomes, mindfulness is particularly relevant, as Hoffmann and Geisler^18^ highlighted the importance of acceptance in reducing stressor threat perceptions.

Guthier et al.^11^ emphasized the importance of simultaneously analyzing stressor effects and strain effects in order to disentangle the mechanisms between job stressors and (psychological) well-being . The authors also pointed out the risk of a vicious cycle between job stressors and mental health (e.g. burnout) when both effects are present simultaneously. By delving into these aspects, our study seeks to make a meaningful contribution to the evolution of stress theory, both in a general sense and specifically in the context of student populations. This research has the potential to pave the way for more effective mental health interventions tailored to the unique needs of students.

The Study Demand-Resources (SD-R) model^6^ is derived from the well-established JD-R model. Lesener et al.^6^ emphasized the necessity of future studies to validate this framework, particularly how study demands and resources correlate with various health outcomes through both health-impairment and motivational pathways.. This is supported by Koob et al.^19^ who highlighted the importance of investigating whether the assumptions of the SD-R also hold under the dynamics of the COVID-19 pandemic in Germany, although, in their case, the research focus was on study engagement. Furthermore, they suggested that more complex relationships could be investigated using structural equation modeling (SEM) models. With our study, we contribute to these calls as we analyzed perceived stressor and strain effects in a cross-lagged panel design with a time lag of 1 year, thus bridging the gap of the lack of longitudinal studies among university students^20^.

We extend the SD-R model by including not only one of the most common outcomes, emotional exhaustion, but also a number of positive and negative indicators of well-being. This is in line with the call of the Okanagan Charter^8^ to study and support well-being at universities. Therefore, we aim first to examine how perceived study demands (workload and work complexity) are reciprocally related to mental health outcomes (emotional exhaustion, well-being, and depression) over time, and second to identify potential moderators for both the stressor, as well as the strain effects.

## Theoretical framework

### Demands-resources framework and the stressor effect

The JD-R theory^10^ and the SD-R model^6^ distinguish between demands and resources. A key proposition of these models is that demands deplete resources, as they require effort and initiate a process that impairs health. This is the process we refer to as the *stressor effect*, which has been widely studied. Resources, on the other hand, are beneficial for goal attainment and therefore thought to lead to motivational gains. Furthermore, resources can buffer the negative effects of demands on exhaustion^10^.

Since most studies using the JD-R and SD-R model focus on emotional exhaustion/burnout in the health-impairment path and engagement in the motivational path, we aim to broaden this perspective by including depression and well-being as general outcomes in addition to study-related emotional exhaustion. First, emotional exhaustion describes the state of students when they feel exhausted and overwhelmed by the demands of studying^21,22^. It is a domain-specific outcome for the context of studying and is an important indicator of students’ mental health.

In contrast, depression, which is a common mental health disorder, is not domain-specific and shows high prevalence rates among university students^4,23^. Depression is considered to be a more distal outcome which develops over a longer period of time, therefore, adding to the understanding of health impairments beyond more proximal outcomes, such as emotional exhaustion. Studies have shown that students with depression are more likely to have lower grades and to be at risk for academic failure^24^. Additionally, students with poor mental health may be more likely to engage in unhealthy behaviors such as substance abuse, unhealthy eating, or physical inactivity^25^. Depression is characterized by a sad mood, loss of interest or pleasure, low-self-esteem, poor concentration, and disturbed sleep^26^. Well-being includes “high satisfaction with life, an abundance of desirable feelings, and low levels of negative feelings”^27^ and has been linked to good life and happiness. A study by Freire et al.^28^ showed that the higher the level of well-being, the higher the level of students’ adaptive coping strategies (e.g. support-seeking) in dealing with academic stressors. Subjective well-being is a positive indicator of mental health and can be viewed as a broader concept, adding value to research on student stress. Also, from a salutogenic perspective, integrating positive states/outcomes into research on student mental health is an interesting endeavor.

These different health outcomes can be related to various resources or stressors according to the JD-R model. We focus on perceived study stressors to shed light on the strain process. Commonly described stressors are workload and work complexity^6,20^. Research in crisis contexts has shown that increased workload and job complexity are often accompanied by work intensification and working overtime^30^, consequences that we would also expect among students during their studies. This is supported by findings from the COVID-19 context, which showed that students’ workload and the amount of time spent studying increased^2,30^. Therefore, we selected perceived workload and work complexity as the core stressors for our analyses. Consistent with the health-impairment path as described in the JD-R and the SD-R model, we propose stressor effects for workload, and work complexity:

*H1 *Workload (T1) is positively related to a) emotional exhaustion and b) depression and negatively related to c) well-being one year later (T2).

*H2 *Work complexity (T1) is positively related to a) emotional exhaustion and b) depression, one year later and negatively related to c) well-being one year later (T2).

### Reversed causation: the strain effect

As Guthier et al.^11^ detail in their meta-analysis, strain effects (effects of mental health on stressors), in contrast to stressor effects, are rarely expected according to stress theories and are not often tested. This includes the methodological risk of misspecified models and biased estimates^11^. However, few studies in the work context reported strain effects or reciprocal effects between stressors and burnout^31–33^. However, in a recent refinement of the JD-R theory, Bakker et al.^34^ explained that job strain has a dual role as an outcome and a predictor of job demands or dysfunctional behaviors. Thus, when an individual experiences strain in the form of emotional exhaustion, anxiety, or depressive symptoms, their energy resources are being depleted, and dysfunctional coping becomes more likely^34^. Then, after several days, weeks, or even months and years, the dysfunctional coping behavior creates additional job demands and consequently leads to increased job strain, with potential loss spirals^34^. The literature on these complex relations between student mental health and study stressors is even more scarce than in the job context, therefore this study contributes to bridging this gap. The meta-analysis by Guthier et al.^11^ found both stressor and strain effects. However, the strain effect of burnout on stressors was markedly stronger.

Guthier et al.^11^ suggest four distinct explanations for strain effects in the work context that we transfer to students, which were labeled the drift and refugee hypotheses, the stressor creation and the stressor perception hypotheses. The drift hypothesis suggests a downward selection process, as employees with poor health might end up in less favorable jobs or even get unemployed. In contrast, the refugee hypothesis suggests a voluntary choice to look for jobs with less stressors. For the student context, these two explanations seem not applicable, despite considering potential dropouts.

According to the stressor creation hypothesis^35^, exhausted students may create a higher workload for themselves. An example of this could be that exhausted students find it harder to manage their work tasks efficiently, leading to accumulating unfinished tasks and an increased workload. This increase in workload is considered "objective" because it can be validated by independent observers noticing an increased number of unfinished tasks. Another factor that might lead to increasing study stressors for students who show symptoms of depression is that depression is typically associated with worry/rumination^36^. Hence, a student might try to analyze a complex problem/task in detail and thereby increase workload or work complexity.

On the other hand, the stressor perception hypothesis^31^ proposes that strain-effects occur because exhausted students perceive higher levels of stressors, even when facing the same amount of objective level of stressors. In other words, there are changes in how students evaluate their study conditions. This hypothesis is supported by findings that individuals with high levels of negative affectivity, a common symptom of strain, tend to interpret ambiguous stimuli more negatively^37^. Similarly, emotional exhaustion has been correlated with a heightened attention to negative or dysphoric stimuli^38^. Therefore, exhausted students may over time perceive their environment more negatively and report increased stressors. Other scholars have described this as the gloomy perception mechanism as burned-out individuals perceive the same task characteristics as gloomier across time^39^. Conversely, healthy students (with high levels of well-being) might perceive study characteristics as rosier over time – this has been referred to as the rosy perception mechanism^34^. Further empirical support for strain effects were reported by Mash et al.^41^ by showing that job satisfaction was negatively related to demands at a later time point.

*H3* a) Emotional exhaustion and b) depression are positively, and c) well-being (T1) is negatively related to workload (T2) one year later (T2).

*H4* a) Emotional exhaustion and b) depression are positively and c) well-being is negatively related to work complexity one year later (T2).

#### Moderating effects of resources in the stressor-strain relationships

According to the JD-R model, resources can moderate (i.e. buffer) the negative effects of stressors on exhaustion^10^. However, studies analyzing possible interaction effects within the JD-R have been inconclusive^21,42,43^. Guthier et al.^11^ did not find a moderating effect of job control on the stressor effect when they used standardized country-level data for the moderators. They had hypothesized that job control might act as a safety signal^44^ and, like other authors^45^ suggested that individual resources should be examined as possible moderators in future research. We propose that social support as a structural resource and mindfulness as a personal resource may act as safety signals that buffer the negative effects of study stressors on various mental health outcomes. Social support is a commonly studied construct in the context of JD-R^21^, so we chose to examine the context-specific version^20^—the social support of both lecturers and peers. We argue that social support can make students feel more prepared to face challenging demands during their studies. Therefore, it could buffer the effect of study stressors on mental health outcomes.

*H5* Social support (T1) buffers the stressor effect of workload on a) emotional exhaustion, b) depression, and c) well-being (T2).

*H6* Social support (T1) buffers the stressor effect of work complexity on a) emotional exhaustion, b) depression, and c) well-being (T2).

Mindfulness, which refers to the “awareness of one’s internal states and surroundings”^46^, has not been studied as frequently within the JD-R^47–50^ or SD-R frameworks. Therefore, its integration into the current study helps to expand our understanding of the role of different personal resources within the stressor and strain relationships. As shown by Grover et al.^47^ in their study of Australian nurses, mindfulness was positively associated with employees’ positive job perceptions and use of job resources. This may then influence the way they experience stress. Previous research has shown that mindfulness is negatively associated with various mental health outcomes (e.g., depression) as it is theorized to protect against the negative effects of stress^50,51^ However, there have also been conflicting findings. In a study by Borden^52^ mindfulness magnified the effects of demands on burnout instead of buffering them. However, this seems to be a very exceptional finding. In contrast in a study by Fisher et al.^49^, mindfulness buffered the effects of workload on strain. Therefore, we would like to contribute to a better understanding of the benefits and limitations of mindfulness and propose the following hypotheses^53^. Since the assumption in line with the theorizing in the frame of the JD-R would be to that mindfulness is considered a resource, we deduce hypotheses accordingly.

*H7* Mindfulness (T1) buffers the stressor effect of workload on a) emotional exhaustion, b) depression, and c) well-being (T2).

*H8* Mindfulness (T1) buffers the stressor effect of work complexity on a) emotional exhaustion, b) depression, and d) well-being (T2).

#### Moderating effects of resources in the strain-stressor relationships

Although it has not been the main focus of stress theories, some studies have called for the investigation of potential moderating factors of the strain effect^11,54^. Finding evidence of moderating effects could explain under what circumstances such strain effects occur^54^. Again, the safety signal hypothesis from the work context^11^ can be applied to the student context: The assumption is that students who already feel emotionally exhausted perceive study stressors as a threat to their already compromised mental health, leading them to perceive stressors as more severe. Social support and mindfulness could act as safety signals, buffering the perceived threat of study stressors to students’ mental health and reducing exaggeratedly perceived study stressors. This hypothesis is consistent with findings in the work context, where job support and job control were shown to act as safety signals in the strain process^11^.

Conversely, resources may enhance the effect of well-being on perceived stressors. When students have high levels of well-being, they can build up resources. It might be easier for them to benefit from structural resources such as social support and to be mindful of their positive state, making them perceive study stressors as easier to cope with. Furthermore, the concept of mindfulness involves perceiving the present moment without judgment. This approach could lead to a scenario where a student may not assess a given situation as a potential source of stress. The selection of social support and mindfulness as potential moderators of the strain-stressor relationship follows the same line of reasoning as the selection of moderators of the stressor-strain relationships, since in both cases, resources buffer against negative perceptions of either stress or health status.

*H9* Social support (T1) buffers the strain effect of a) emotional exhaustion, b) depression, and c) well-being on workload (T2).

*H10* Social support (T1) buffers the strain effect of a) emotional exhaustion, b) depression, and c) well-being on work complexity (T2).

*H11* Mindfulness (T1) buffers the strain effect of a) emotional exhaustion, b) depression, and c) well-being on workload (T2).

*H12* Mindfulness buffers the strain effect of a) emotional exhaustion, b) depression, and c) well-being on work complexity (T2).

## Methods

### Data collection and study design

To test our hypotheses, we conducted a two-wave panel study with a one-year lag. All students at the University of Johannes Gutenberg University Mainz were invited by e-mail to participate in an online health survey as part of the annual health survey taking place. The first survey period took place in June 2020 (T1)^55^, and the second in June 2021 (T2), at a German university in the middle of the semester while classes take place and before the typical exam period starts. Several reminder e-mails were sent, and various channels were used to promote the survey. The topics assessed were sociodemographics, study conditions, psychological resources, health-related behaviors, and health outcomes. Matching codes were used to identify participants who participated at both time points. Informed consent by participants was obtained. Ethical Clearance was provided by the Institute of Psychology of Johannes Gutenberg University Mainz (2020-JGU-psychEK-S008). The study was performed in accordance with the Code of Ethics of the World Medical Association (Declaration of Helsinki) for experiments involving humans and the Ethical Principles and Guidelines for the Protection of Human Subjects of Research by the American Psychological Association (APA).

### Sample

Initially, 3066 university students completed the online questionnaire in 2020, and 1438 in 2021. Initial calculations conducted with Soper's^56^ SEM sample size calculator indicated that a minimum sample size of 229 would be necessary to achieve sufficient statistical power for the analyses. This determination was grounded in the expectation of a small-to-medium effect size (0.25), the presence of five latent variables and 10 observed variables, and the aim for a statistical power of 0.8, with a significance level set at 0.05. A total of 264 university students participated at both time points. Among these, the majority (*n* = 217; 82.2%) of participants was female, 17.4% (*n* = 46) were male, and 0.5% (*n* = 2) identified as diverse. The mean age was 22.8 (*SD* = 3.4) years. Students from all faculties participated. A large proportion of participants were from the Faculty of Social Sciences, Media, and Sports (*n* = 51; 19.3%), from the Faculty of Philosophy and Philology (*n* = 41; 15.5%), and in the field of education/pedagogy (n = 44; 16.7%). A dropout analysis revealed that no specific group of students (based on gender, age, field of study, emotional exhaustion T1, depression T1, or well-being T1) was significantly more likely to drop out from the second survey after completing the first survey.

### Measures

***Emotional exhaustion*** was assessed using five items from the German version of the Maslach Burnout Inventory for students [MBI-S]^57^. An example item is “I feel emotionally drained by my studies.” Items were answered on a seven-point Likert-type scale from “never” (1) to “always” (7). Cronbach’s *α* was 0.91 at T1, and 0.92 at T2.

***Depression*** was assessed using the Patient Health Questionnaire 9 (PHQ-9), which measures nine symptoms of major depression over the past 14 days^58^. An example item is: “Little interest or pleasure in doing things”, with a response scale ranging from “not at all” (0) to “almost every day” (3). Cronbach’s *α* was 0.85 at T1, and 0.88 at T2.

***Well-being*** was measured using the German WHO’s Five Well-being Index^59^, on a 6-point Likert scale ranging from “at no time” (1) to “all the time” (5). An example of the five items is ‘The last two weeks I was happy and in a good mood.’ Cronbach’s *α* was 0.85 at T1, and 0.88 at T2.

***Workload/quantitative demand****s* were measured with one item from the COPSOQ^60^ adapted to the student context: “Do you have enough time to complete all your study-related tasks?” Response options were ranging from “never/hardly ever” (1) to “always” (5). To capture quantitative demands, the item was recoded.

***Work complexity*** was assessed using one item from the Berliner Anforderungs Ressourcen Inventar für Studierende (BARI-S) [*Berlin Demands Resources Inventory*]^20^: “It happens that my studies are too difficult.” Responses were ranging from “never/hardly” (1) to “always” (5).

***Social support*** from lecturers/peers was measured using the Salutogenic Subjective Job-Analysis Questionnaire developed by Rimann and Udris^61^, with three items for lecturers and three for peers. One example question was, “How much can you rely on your lecturers/fellow students when you face problems during your studies/while studying?” Answers could range from “not at all” (1) to “completely” (5). Cronbach’s *α* was 0.86 at T1, and 0.90 at T2 for support from lecturers, and 0.90 at both time points for support from peers.

***Mindfulness*** was assessed using the 14-item Freiburg Mindfulness Inventory (FMI)^62^. An example item is “I am open to the experience of the present moment.” Responses ranged from “rarely” (1) to “almost always” (4). Cronbach’s *α* was 0.85 at both time points.

### Measurement invariance and analyses

Prior to hypotheses testing, we found evidence for assuming measurement invariance between the two time points for emotional exhaustion, depression, and well-being (see Table S1 in the online supplement). Descriptives of the mental health outcomes and according cut-offs were analyzed. PHQ9 and WHO5 are usually used as sum-scores, for the structural equation models, mean scores were calculated for PHQ9 and WHO5. We performed separate cross-lagged panel structural equation models (see notes under Tables 2, and 3 for further information) for each health outcome in combination with each study stressor (workload, and work complexity). We then performed additional cross-lagged panel analyses, including the interactions effects (separately for each moderator). Statistical significance was defined as *p* < 0.05. The analyses were performed using SPSS 23 and Mplus 7.3^63^.

## Results

Descriptives, Cronbach’s alpha values, and correlations are displayed in Table 1. Results of the cross-lagged structural equation models are presented in Tables 2, and 3.

**Table 1:** Means, standard deviations, and bivariate correlations between study variables.

Variable	*M*	*SD*	1	2	3	4	5	6	7	8	9	10	11	12	13	14	T(ΔM)
1. Emotional exhaustion T1	4.31	1.57	(.91)														1.99*
2. Depression T1	0.96	0.58	.53**	(.85)													− 2.39*
3. Well-being T1	2.38	1.02	− .54**	− .72**	(.85)												0.91
4. Workload T1	3.31	1.27	.55**	.25**	− .26**	–											0.64
5. Work complexity T1	2.74	1.13	.55**	.37**	− .32**	.45**											1.17
6. Social support: lecturer T1	3.34	0.86	− .42**	− .22**	.26**	− .28**	− .33**	(.86)									
7. Social support: peers T1	3.57	1.02	− .22**	− .21**	.23**	–.12	− .08	.26**	(.90)								
8. Mindfulness T1	2.57	0.48	− .29**	− .52**	.46**	− .08	− .14*	.16*	.23**	(.85)							
9. Emotional exhaustion T2	4.12	1.50	.45**	.45**	− .30**	.35**	.43**	− .28**	− .02	− .31**	(.92)						
10. Depression T2	1.04	0.63	.32**	.63**	− .42**	.14*	.26**	− .14*	− .10	− .45**	.53**	(.88)					
11. Well-being T2	2.31	1.03	− .38**	− .55**	.49**	− .18**	− .28**	.18**	.17**	.50**	− .48**	− .73**	− .53**	(.88)			
12. Workload T2	3.26	1.27	.28**	.24**	− .09	.43**	.40**	− .27**	.00	− .02	.53**	.24**	.24**	− .18**	–		
13. Work complexity T2	2.64	1.18	.28**	.25**	− .07	.27**	.50**	− .24**	− .05	− .15*	.59**	.41**	.26**	− .27**	.46**	–	

*M* and *SD* represent mean and standard deviation, respectively. Cronbach’s *α* is presented in parentheses; ** *p* < .01, * *p* < .05. T(ΔM) = T-Test for dependent samples for mean differences between T1 and T2.

**Table 2:** Results of (moderated) cross-lagged panel analyses for workload.

	Emotional Exhaustion			Depression			Well-Being		
Hypotheses	1a/3a	5a/9a	7a/11a	1b/3b	5b/9b	7b/11b	1c/3c	5c/9c	7c/11c
Autoregressive effects									
Workload	.39 (.07)***	.39 (.07)***	.38 (.07)***	.39 (.06)***	.38 (.07)***	.39 (.07)***	.45 (.06)***	.42 (.07)***	.46 (.07)***
Outcome	.36 (.08)***	.33 (.07)***	.28 (.08)**	.77 (.06)***	.80 (.07)***	.68 (.09)***	.52 (.07)***	.49 (.07)***	.25 (.09)**
Synchronous effects									
Workload T1 ↔ outcome T1	.67 (.07)***	.66 (.07)***	.66 (.07)***	.23 (.05)***	.23 (.05)***	.23 (.06)***	− .35 (.08)***	−. 36 (.09)***	− .37 (.09)***
Workload T2 ↔ outcome T2	.59 (.08)***	.53 (.07)***	.60 (.07)***	.09 (.04)*	.09 (.04)*	.10 (.04)*	− .20 (.08)*	− .16 (.07)*	− .15 (.07)*
Cross-lagged effects									
Workload T1 → Outcome T2	.13 (.07)*	.12 (.06)*	.15 (.07)*	− .03 (.03)	− .03 (.03)	− .02 (.02)	− .02 (.06)	− .02 (.05)	− .04 (.05)
Outcome T1 → Workload T2	.10 (.09)	.06 (.09)	.13 (.09)	.33 (.14)*	.29 (.15) +	.46 (.21)*	.04 (.08)	.08 (.09)	.05 (.11)
Moderator → Workload T2									
Support – lecturer		− .31 (.12)**			− .29 (.11)**			− .37 (.11)**	
Support – peers		.14 (.08) +			.20 (.08)*			.16 (.08) +	
Mindfulness			.07 (.25)			.50 (.29) +			− .01 (.29)
Moderator → Outcome T2									
Support – lecturer		− .01 (.25)			− .14 (.13)			− .06 (.25)	
Support – peers		.05 (.17)			.02 (.12)			.12 (.19)	
Mindfulness			− .40 (.51)			− .17 (.23)			.77 (.36)*
Interactions									
Workload × Support—lecturer		− .05 (.07)			.05 (.03)			.03 (.07)	
Outcome × Support—lecturer		− .21 (.10)*			− .24 (.20)			.00 (.12)	
Workload × Support—peers		− .03 (.07)			.01 (.03)			− .01 (.06)	
Outcome × Support – peers		.02 (.05)			− .20 (.13)			.09 (.09)	
Workload × Mindfulness			− .04 (.14)			− .01 (.05)			.10 (.08)
Outcome × Mindfulness			− .31 (.23)			− .47 (.40)			.40 (.20)*

Unstandardized estimates with standard errors in brackets; Emotional Exhaustion, Well-Being, and Social Support by Lecturers, and Peers were modeled as latent constructs based on single items. Depression and Mindfulness were latently modeled using three, and four parcels, respectively. Workload was measured with a single item, and thus included as manifest variable. Interaction effects were latently modeled. + *p* < .10, * *p* < .05, ** *p* < .01, *** *p* < .001.

**Table 3:** Results of (moderated) cross-lagged panel analyses for work complexity.

	Emotional Exhaustion			Depression			Well-Being		
Hypotheses	2a/4a	6a/10a	8a/12a	2b/4b	6b/10b	8b/12b	2d/4d	6d/10d	8d/12d
Autoregressive effects									
Complexity	.45 (.08)***	.44 (.11)***	.47 (.11)***	.49 (.07)***	.40 (.09)***	.44 (.09)***	.52 (.06)***	.47 (.09)***	.51 (.08)***
Outcome	.29 (.09)**	.29 (.08)**	.24 (.09)**	.77 (.06)***	.80 (.07)***	.68 (.10)***	.48 (.07)***	.45 (.07)***	.23 (.09)**
Synchronous effects									
Complexity T1 ↔ outcome T1	.71 (.06)***	.70 (.06)***	.70 (.06)***	.23 (.05)***	.30 (.06)***	.30 (.06)***	− .40 (.08)***	− .40 (.08)***	− .40 (.08)***
Complexity T2 ↔ outcome T2	.70 (.07)***	.69 (.09)***	.72 (.09)***	.09 (.04)*	.21 (.04)***	.23 (.04)***	− .23 (.07)**	− .23 (.08)**	− .17 (.08)*
Cross-lagged effects									
Complexity T1 → Outcome T2	.22 (.07)**	.19 (.08)*	.22 (.08)**	− .03 (.03)	− .02 (.03)	− .01 (.03)	− .13 (.05)*	− .12 (.06)*	− .10 (.05)*
Outcome T1 → Complexity T2	.04 (.10)	− .01 (.11)	.02 (.11)	.33 (.14)*	.27 (17)	.31 (.22)	.13 (.08)	.16 (.09) +	.25 (.10)*
Moderator → Complexity T2									
Support – lecturer		− .19 (.10) +			− .16 (.11)			− .22 (.10)*	
Support – peers		.05 (.08)			.08 (.08)			− .01 (.09)	
Mindfulness			− .11 (.22)			.07 (.29)			− .50 (.25)*
Moderator → Outcome T2									
Support – lecturer		.02 (.24)			.03 (.11)			− .15 (.22)	
Support – peers		.11 (.15)			− .05 (.11)			.19 (.16)	
Mindfulness			− .73 (.47)			− .23 (.22)			.93 (.43)*
Interactions									
Complexity × Support—lecturer		− .06 (.07)			− .01 (.03)			.04 (.06)	
Outcome × Support—lecturer		.07 (.09)			.01 (.15)			− .07 (.08)	
Complexity × Support—peers		− .00 (.05)			.03 (.03)			− .03 (.05)	
Outcome × Support – peers		.01 (.07)			.01 (.14)			− .10 (.08)	
Complexity × Mindfulness			.09 (.13)			.01 (.06)			.04 (.11)
Outcome × Mindfulness			.22 (.18)			.18 (.28)			− .20 (.15)

Unstandardized estimates with standard errors in brackets; Emotional Exhaustion, Well-Being, and Social Support by Lecturers, and Peers were modeled as latent constructs based on single items. Depression and Mindfulness were latently modeled using three, and four parcels, respectively. Work Complexity was measured with a single item, and thus included as manifest variable. Interaction effects were latently modeled. + *p* < .10, * *p* < .05, ** *p* < .01, *** *p* < .001.

According to the PHQ9, 34.8% (n = 88) of students reached the cut-off for depression at T1 and 41.7% (n = 103) at T2^64^. According to the WHO5, 22.3% (n = 57) of students reached the cut-off for depression at T1 and 26.2% (n = 64) at T2^65^. We furthermore tested whether stressors or strains changed between T1, and T2. The results are presented in the last columns in Table 1. We could observe a small significant decline in emotional exhaustion, and an increase in depressive symptoms. Neither for well-being, nor for workload or work complexity mean differences over time reached statistical significance.

### Workload and mental health

The cross-lagged panel analyses revealed that workload (T1) predicted emotional exhaustion (T2), but not vice versa, confirming H1a and rejecting H3a. Workload (T1) did not predict depression (T2), failing to support H1b, whereas depression (T1) predicted workload (T2), supporting H3b. Neither H1c nor H3c was supported because workload, and well-being were not related in either direction.

### Work complexity and mental health

Work complexity (T1) predicted emotional exhaustion (T2), but not vice versa, confirming H2a and rejecting H4a. H2b could not be supported, as work complexity (T1) did not predict depression (T2). However, depression (T1) was a predictor of work complexity (T2), confirming H4b. H2c was confirmed as work complexity (T1) predicted well-being (T2). On the other hand, H4c was not supported because well-being (T1) was not a predictor of work complexity (T2).

### Interaction effects

**Figure 1:**
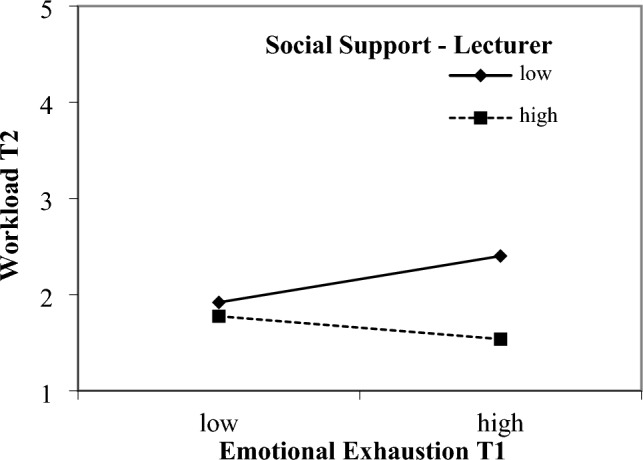
Interaction effect of emotional exhaustion and social support by lecturers. Interaction Effect of Emotional Exhaustion at T1 and Social Support by Lectures in Predicting Workload T2. Simple slope for low values of the moderator (b = .23, T = 2.058, p = .041), and high values of the moderator (b = − .12, T = − .853, p = .395).

**Figure 2:**
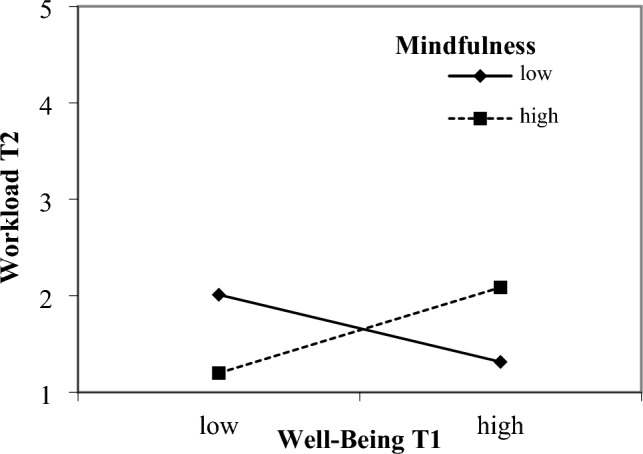
Interaction effect of well-being and mindfulness. Interaction Effect of Well-Being at T1 and Mindfulness in Predicting Workload T2. Simple slope for low values of the moderator (b = − .35, T = − 1.505, p = .134), and high values of the moderator (b = .44, T = 1.972, p = .050).

Almost all hypotheses regarding interaction effects (H6-12) were rejected, with one exception. H9a was supported in that social support by lecturers moderated the strain effect of emotional exhaustion on workload (Fig. 1). A simple slope test revealed a significant positive effect for low values of social support (*b* = 0.23, *T* = 2.058, *p* = 0.041). Although we found a significant interaction effect between well-being and mindfulness on workload, further analyses revealed that simple slopes were not significant (Fig. 2).

## Discussion

The current study was one of the first to simultaneously examine stressor and strain effects (in combination with resources) among university students, thereby shedding new light on current stress theory developments. The two-wave 1-year cross-lagged panel study revealed several stressor effects: perceived workload and work complexity predicted emotional exhaustion, and perceived work complexity predicted well-being. Our results partially supported the notion that strain effects play an essential role in the student stress dynamics, as depression predicted perceived workload and work complexity. When the social support of lecturers was low, it increased the strain effect of emotional exhaustion on perceived workload. Contrary to our expectations, mindfulness and peer social support did not enhance other effects.

### Theoretical contribution

Our study contributes to the stress literature by examining the reciprocal effects of stressors and strain in a student sample. We answered the call to verify the SD-R framework^6^ with common study stressors (perceived workload and work complexity) and even extended the framework by examining a variety of mental health outcomes and their conditional reciprocal relationships with specific study stressors. Furthermore, we were able to transfer findings from the work context^11^ to the study context. Confirming stress theories, such as the JD-R model, we found a stressor effect of perceived workload and work complexity on emotional exhaustion. This is consistent with numerous studies that found positive associations between job or study demands and burnout^6,66,67^. In addition, we found a stressor effect of perceived work complexity on well-being. However, we did not find a stressor effect of perceived workload or work complexity on depression. This may be because depression is a more distal outcome. Another explanation might be the long time lag of one year. However, caution should be exercised in drawing premature conclusions about the relationship between study stressors and depression, as different time lags and different operationalization of stressors may lead to different effects. Our findings contrast with a study by Hatch et al.^68^, which showed that job stressors are associated with depression and burnout symptoms over time. The authors noted the need to study burnout, depression, and job stressors simultaneously when assessing the mental health of employees^67^. It also needs to be taken into account that we only studied very specific aspects of study stressors (perceived time available and complexity of course work), so that our findings do not allow for generalization of study stressor effects.

Contrary to our expectation, we did not find an effect of emotional exhaustion on perceived study stressors when potential moderators were not taken into account. This may be because emotional exhaustion is a more temporal phenomenon that can vary from day to day and does not influence study stressors one year later. In a study by Guthier et al.^11^, burnout had a strain effect in contrast to our study, where emotional exhaustion showed almost no strain effect. Conversely, our hypotheses regarding the strain effect of depression were confirmed. These results indicate that students who experience symptoms of depression are doubly burdened, as they experience more severe study stressors, in this case increased perceived workload and work complexity as a result.

The identified strain effects raise the question of the potential underlying mechanisms. We hypothesized a stressor creation or stressor perception effect (gloomy effect)^31^. Due to a lack of objective data, we cannot conclude whether students actually experience more study stressors or whether their perception of study stressors is higher. Either way, it is important to prevent these consequences for students, as both outcomes could have a negative impact on mental health in the long run. Since the data of the current study rely on subjective perception it might seem plausible to conclude that the gloomy perception explains the strain effect. The stressor creation effect could only be adequately verified with objective data. In favor of our assumption of a stressor creation effect is the fact that subjectively measured stressors correspond strongly with objective measures as indicated by previous research^69^, therefore, it seems reasonable to assume that at least part of the effect of strain on workload is due to an actual increase in workload.

The stressor and strain effects of our study highlight the danger of a potential vicious cycle. As our results show, the study stressors perceived workload and work complexity predict emotional exhaustion. Other studies show that emotional exhaustion is related to depression, although causality cannot be concluded^70^. According to our findings, strain, such as depression, may increase the perception of study stressors (workload and work complexity), which in turn leads to further increased symptoms of emotional exhaustion. If this cycle is not adequately interrupted with supportive resources or recovery periods^11^, student mental health problems will persist or even increase. These expected mechanisms are also consistent with COR, which suggests that people who lack resources are more likely to experience adverse outcomes^12^. People with fewer resources are also more likely to be primarily affected by crises or stressors than people with more resources. As a result, initial setbacks can be detrimental and lead to rapid loss spirals^71^. Experiencing strain usually goes along with losing resources. As a consequence, it could be that future stressors are perceived as more difficult to manage since there are fewer resources available to cope with.

#### Role of moderators

No interaction effects between the resources (social support and mindfulness) and stressors were found. Therefore, the safety signal hypothesis^43^ had to be rejected. This finding is consistent with the meta-analysis of Guthier et al.^11^, in which they found only interaction effects for the strain effect. However, this contrasts with other studies that have demonstrated how different resources could exert a buffering effect^10,42,47^. The JD-R framework suggests interaction effects between demands and resources^42^, but not between strain and resources. However, this is an important avenue for future research within the JD-R or SD-R research landscape^6^.

Despite that we had to reject most of our hypotheses regarding moderators of strain effects, we can report an interesting finding. A strain effect of emotional exhaustion on workload was only detected when social support by lecturers was low. This finding underscores how a lack of social support could potentially have long-lasting detrimental effects on emotionally exhausted students. Consistent with the previously proposed safety signal hypothesis^11^, students struggling with emotional exhaustion perceive study stressors as a potential threat to their already compromised mental health when they lack social support. Since we only found one moderating effect of social support by lecturers, this finding should be interpreted cautiously, however. A possible explanation for the lack of further moderating effects could be derived from the triple match theory^72^. It suggests that a match of stressor, strain, and a moderator is needed to find interaction effects. Mindfulness and social support may not have been the best matching resources in relation to workload, and work complexity. This does not preclude other resources from acting as moderators.

### Limitations and future directions

A limitation of the present study is the relatively long time span of one year, which may not have captured more proximal effects. For instance, it might be that strain effect of emotional exhaustion show after a few days or weeks only which could be analyzed in diary studies. In addition, the experience of study demands often occurs in cycles, with periods of high workload just before exams and relatively low workload at the beginning of the semester; it might be interesting for future research to identify the optimal study interval. Even longer time intervals might not be promising in terms of dropout risks as there is a quite high level of fluctuation among students at universities. Three time points would have been better for making causal inferences^73^ and a reciprocal continuous time perspective could shed additional light on the stressor-strain debate^11^. Furthermore, we cannot explain the mechanisms behind stressor and strain effects since we did not use objective measures. Future studies could strengthen their investigations by using objective measures to reduce potential common method bias. In addition, the study stressors could be measured more comprehensively by including more items that cover a wider spectrum. Since the inclusion of study participants was based on non-probability sampling, the representativeness of our sample can be questioned. Also, the high proportion of female participants limits the generalizability of the findings. However, we included students from a variety of disciplines. When interpreting our data, the special circumstances during which the study took place (in the middle of the pandemic) need to be taken into account. Since the results might be specific to these pandemic circumstances, replication of similar analyses is required.

One way to open avenues for future research would be to include more favorable outcomes, such as engagement and various resources, and determine whether these are similarly reciprocally related. Another way to assess how to mitigate stressor and strain effects might be to examine other potential moderators, such as self-efficacy or autonomy. Similarly, the influence of home demands and resources^66^ on student outcomes deserves further investigation.

### Practical implications

The study underscores the importance of structured health promotion and mental health initiatives on university campuses, aligning with the Okanagan Charter^8^. Strain effects highlight the need for supporting students with mental health issues to prevent long-lasting consequences. It is important to avoid a vicious cycle in which study stressors, such as perceived workload and work complexity, lead to emotional exhaustion, and emotional exhaustion can lead or be accompanied by depression, which can lead to further exposure to study stressors. Ultimately, persistent strain and high study demands can lead to dropout. Student counselors and educators need to be aware of these effects to help their students adequately. Since one possible explanation for the strain effect is the previously described gloomy perception effect, strategies such as cognitive reappraisal that assist students in appropriately assessing their study demands could be beneficial^74^. Furthermore, active coping strategies that should be encouraged have been shown to influence emotional exhaustion due to overload^75^. In addition, students could be encouraged to show study crafting^76^ which could help them become aware of and utilize available social resources in their study context but also transform demands to challenges.

Finally, only one finding of our study results demonstrated the potential of social support by lecturers for students. We found that students who receive low levels of social support from lecturers experience a strain effect of emotional exhaustion on workload. Therefore, it is crucial that lecturers demonstrate their support by being available, especially to students who are already burdened, and advising them on how they can get further help. Although our study only revealed one result showing the negative effect of low social support and does not allow to draw comprehensive conclusions in this regard, the important role of social support in general is quite evident. Other authors have pointed out how supervisor support can target subjectively experienced workload by reducing perceived demands, for example, by showing students how to reframe stressful situations^9^. Similarly, the beneficial effects of social support may be magnified by its direct impact on mental health, as demonstrated by other research^77^.

## Conclusion

Our study contributes to the current literature by demonstrating how study stressors and student strain are reciprocally related over time. We provide further evidence that previously understudied strain effects exist and can be moderated. In doing so, we verify and extend current stress theories. These findings highlight the need and currently developing trend^10^ to extend stress theories, such as the JD-R of SD-R, by examining stressor and strain effects simultaneously. We encourage future research among university students to consider strain effects and identify further moderators that may mitigate these effects. In addition, practical implications can be drawn regarding how to support students with mental health problems, such as reducing study stressors like perceived workload and promoting lecturer social support to create a health-promoting campus environment.

### Supplementary Information

Supplementary Table 1.

## Data Availability

The datasets generated during and/or analyzed during the current study are available from the corresponding author on reasonable request.
