# Supplementary material for: Conditional reciprocal stressor–strain effects in university students: a cross-lagged panel study in Germany
Source: Sci Rep. 2024 Mar 23;14:6952. doi: 10.1038/s41598-024-57486-0 (PMC10960860; doi:10.1038/s41598-024-57486-0)
Supplement: Supplementary file 1 — Supplementary Table 1. [file 41598_2024_57486_MOESM1_ESM.pdf]

Online Supplement for

Conditional Reciprocal Stressor-Strain Effects in University Students: A Cross-Lagged Panel Study in Germany

Table S1

Longitudinal Measurement Invariance of Emotional Exhaustion, Depressive Symptoms, and Well-Being

	χ2 (df)	Δχ2 (df)	CFI	TLI	RMSEA	SRMR	ΔCFI	ΔTLI	ΔRMSEA	ΔSRMR
**Emotional Exhaustion**										
Configural Invariance	96.39(28)		.964	.943	.097	.029				
Metric Invariance	107.55(33)	11.16(5)	.961	.947	.093	.045	.003	.004	.004	.016
**Depressive Symptoms**										
Configural Invariance	261.59(124)		.933	.918	.065	.056				
Metric Invariance	274.19(133)	9.60(9)	.932	.921	.064	.060	.001	.003	.001	.004
**Well-Being**										
Configural Invariance	48.91(28)	0.53(5)	.984	.974	.053	.037				
Metric Invariance	49.44(33)		.987	.983	.044	.037	.003	.009	.009	.000

Note. *** $p < .001$, ** $p < .01$, * $p < .05$, As Anxiety was measured with two items, a test on measurement invariance cannot be performed.
